# Synthesis, Physicochemical and Biological Study of Gallium-68- and Lutetium-177-Labeled VEGF-A_165_/NRP-1 Complex Inhibitors Based on Peptide A7R and Branched Peptidomimetic

**DOI:** 10.3390/pharmaceutics14010100

**Published:** 2022-01-01

**Authors:** Katarzyna Masłowska, Ewa Witkowska, Dagmara Tymecka, Paweł Krzysztof Halik, Aleksandra Misicka, Ewa Gniazdowska

**Affiliations:** 1 Centre of Radiochemistry and Nuclear Chemistry, Institute of Nuclear Chemistry and Technology, Dorodna 16, 03-195 Warsaw, Poland; p.halik@ichtj.waw.pl (P.K.H.); e.gniazdowska@ichtj.waw.pl (E.G.); 2 Faculty of Chemistry, University of Warsaw, Pasteura 1, 02-093 Warsaw, Poland; ewawit@chem.uw.edu.pl (E.W.); dulok@chem.uw.edu.pl (D.T.)

**Keywords:** ^68^Ga/^177^Lu-radiopharmaceuticals, cancer therapy, Neuropilin-1, VEGF_165_/NRP-1 complex inhibitor, A7R peptide, peptidomimetics, angiogenesis, tyrosine kinase inhibitor

## Abstract

Neuropilin-1 (NRP-1) is a surface receptor found on many types of cancer cells. The overexpression of NRP-1 and its interaction with vascular endothelial growth factor-165 (VEGF_165_) are associated with tumor growth and metastasis. Therefore, compounds that block the VEGF_165_/NRP-1 interaction represent a promising strategy to image and treat NRP-1-related pathologies. The aim of the presented work was to design and synthesize radioconjugates of two known peptide-type inhibitors of the VEGF_165_/NRP-1 complex: A7R peptide and its shorter analog, the branched peptidomimetic Lys(hArg)-Dab-Pro-Arg. Both peptide-type inhibitors were coupled to a radionuclide chelator (DOTA) via a linker (Ahx) and so radiolabeled with Ga-68 and Lu-177 radionuclides, for diagnostic and therapeutic uses, respectively. The synthesized radioconjugates were tested for their possible use as theranostic-like radiopharmaceuticals for the imaging and therapy of cancers that overexpress NRP-1. The obtained results indicate good efficiency of the radiolabeling reaction and satisfactory stability, at least 3t_1/2_ for the ^68^Ga- and 1t_1/2_ for the ^177^Lu-radiocompounds, in solutions mimicking human body fluids. However, enzymatic degradation of both the studied inhibitors caused insufficient stability of the radiocompounds in human serum, indicating that further modifications are needed to sufficiently stabilize the peptidomimetics with inhibitory properties against VEGF_165_/NRP-1 complex formation.

## 1. Introduction

NRP-1 plays one of the most important roles in the development of angiogenesis, which is the formation of new blood vessels from existing ones. One of the main stages of angiogenesis is the interaction between the pro-angiogenic factor (VEGF-A_165_), its receptor (VEGFR-2), and the NRP-1 co-receptor [1,2,3,4,5,6,7]. NRP-1 acts in two ways: it binds to the pro-angiogenic ligand VEGF-A_165_ through its b1/b2 subdomains, and in parallel, it acts as a co-receptor for VEGFR-2. The resulting ternary VEGF-A_165_/VEGFR-2/NRP-1 complex induces autophosphorylation of the VEGFR-2 tyrosine kinase domains, which influences cell proliferation, differentiation, migration, gene expression, and apoptotic survival of endothelial cells, which ultimately induces angiogenesis [8,9,10]. Many reports also indicate that NRP-1, which is found on many types of cancer cells, lacks catalytic activity and may also serve as a separate receptor for VEGF-A_165_, stimulating tumor growth and metastasis. NRP-1 overexpression may also increase tumor growth and is often associated with poor prognosis, especially in tumors of epithelial origin [3,11,12,13,14,15,16,17,18]. Due to the significant role of NRP-1 in angiogenesis and the relatively well-known mechanisms of the formation and action of the VEGF-A_165_/NRP-1 complex, the design of compounds that block the formation of this complex is an interesting direction in the search for anti-angiogenic and anti-cancer drugs [4,19,20,21,22,23,24,25]. Such angiogenesis inhibitors that target the VEGF-A_165_/VEGFR-2/NRP-1 complex constitute a wide variety of compounds, including anti-VEGF or anti-VEGFR monoclonal antibodies [26,27,28], VEGFR-binding peptides and proteins [23,29,30], small molecular inhibitors of receptor tyrosine kinases of VEGF receptors [22,31,32,33], and various NRP-1-targeting substances such as peptides and peptidomimetics [8,34,35,36,37,38,39,40,41,42,43,44,45,46]. A significant achievement was the identification (by a mutated phage library screening) of a heptapeptide Ala-Thr-Trp-Leu-Pro-Pro-Arg (A7R), which selectively inhibits VEGF_165_ binding to NRP-1 and decreases breast cancer angiogenesis and growth in vivo [35,47]. Further studies of the shortest active fragment of A7R led to a more elaborate branched peptidomimetic, Lys(hArg)-Dab-Pro-Arg, which was a stronger inhibitor of VEGF-A_165_ binding with NRP-1, as determined by an in vitro ELISA assay, and was more stable in human serum compared to A7R [43].

At the same time, angiogenesis inhibitors labeled with diagnostic radionuclides (emitters of gamma or beta plus radiation) or therapeutic radionuclides (emitters of Auger electrons and alpha or beta minus radiation) can serve as diagnostic or therapeutic radiopharmaceuticals, respectively. The diagnostic methods of nuclear medicine can detect diseases at an early stage, much earlier than the accompanying morphological changes that could be detected by classical medicinal diagnosis. Such early and apposite diagnoses strongly promote the effectiveness of consecutive therapy. Importantly, radiocompounds designed from these compounds must maintain their inhibitory activity despite modifications to their chemical structure, such as appending a chelator.

In recent years, targeting overexpressed receptors on tumor cells with radiolabeled peptides has become very important in anticancer therapy [48], so we decided to investigate two novel radioconjugates based on VEGF-A_165_/NRP-1 inhibitors: the A7R peptide and Lys(hArg)-Dab-Pro-Arg peptidomimetic.

The aim of the presented research was the design and synthesis of novel radioconjugates as well as physicochemical characterization of the obtained radiocompounds in terms of the requirements for receptor radiopharmaceuticals.

## 2. Materials and Methods

### 2.1. Materials

Unless otherwise specified, reagents and solvents were obtained from commercial sources and used without further purification. Fmoc-Arg(Pbf)-Wang resin was obtained from Activotec (Cambridge, UK). Amino acids and coupling reagents were purchased from Iris Biotech (Marktredwitz, Germany). DOTA-tris(tBu)-NHS was purchased from CheMatech (Dijon, France). Pooled human serum (HS) was obtained from Innovative Research (Novi, MI, USA). 

Ga-68 radionuclide (emitter β^+^, t_1/2_ = 67.7 min, E_βmax_ = 1.92 MeV) in the form of [^68^Ga]GaCl_3_ in 0.1 M HCl was obtained from a ^68^Ge/^68^Ga generator (Eckert & Ziegler, Germany). Lu-177 radionuclide (emitter β^−^ (76%), t_1/2_ = 6.65 d, E_βmax_ = 0.497 MeV) in the form of [^177^Lu]LuCl_3_ in 0.04 M HCl was purchased from National Centre for Nuclear Research Radioisotope Centre POLATOM, Świerk-Otwock, Poland, at a specific activity ≥ 370 GBq/mg Lu.

Conjugate analyses were performed on a KNAUER RP-HPLC on an analytical Eurospher-100-C-18 column (5 μm, 250 × 4.6 mm). Radioconjugate analyses were performed on a Shimadzu RP-HPLC on a semi-preparative Phenomenex Jupiter Proteo 90Å column (4 μm, 250 × 10 mm) with a Jupiter Proteo precolumn (20 × 2.1 mm) and on an analytical Phenomenex Jupiter 4u Proteo 90Å column (4 μm, 250 × 4.6 mm).

Deionized water was prepared in a Hydrolab water purification system (Hydrolab, Straszyn, Poland).

### 2.2. Methods

#### 2.2.1. Analytical Methods

Conjugates **1** and **2** (compounds with a DOTA chelator) were analyzed by reverse-phase high-pressure liquid chromatography (RP-HPLC) in System 1 or 2. 

**System 1**: RP-HPLC analytical Eurospher-100-C-18 column, 5 μm, 250 × 4.6 mm, solvent A: water with 0.1% trifluoroacetic acid (TFA, *v/v*), solvent B: acetonitrile/water (80:20, *v/v*) with 0.1% TFA (*v/v*), UV/Vis detection at 220 nm, gradient elution: 0–20 min 20 to 70% B, flow 1 mL/min.

**System 1a**: RP-HPLC semi-preparative Nucleosil-300-C18 column, 5 μm, 250 × 8 mm, solvent A: water with 0.1% trifluoroacetic acid (TFA, *v/v*), solvent B: acetonitrile/water (80:20, *v/v*) with 0.1% TFA (*v/v*), UV/Vis detection at 220 nm, gradient elution: 0–5 min 8% B, 5–15 min 8 to 18% B, 15–25 min 18% B, 25–35 min 18 to 35% B, 35–55 min 35% B, 55–60 min 35 to 40% B, 60–65 min 40 to 100% B, flow 2 mL/min.

**System 2**: RP-HPLC analytical Eurospher-100-C-18 column, 5 μm, 250 × 4.6 mm, solvent A: water with 0.1% TFA (*v/v*), solvent B: acetonitrile/water (80:20, *v/v*) with 0.1% TFA (*v/v*), UV/Vis detection at 220 nm, gradient elution: 0–20 min 5 to 60% B, flow 1 mL/min.

**System 2a**: RP-HPLC semi-preparative Nucleosil-300-C18 column, 5 μm, 250 × 8 mm, solvent A: water with 0.1% trifluoroacetic acid (TFA, *v/v*), solvent B: acetonitrile/water (80:20, *v/v*) with 0.1% TFA (*v/v*), UV/Vis detection at 220 nm, gradient elution: 0–15 min 5 to 15% B, 15–45 min 15% B, 45–55 min 15 to 100% B, flow 2 mL/min.

Analyses and purification of the radiopreparations and their cold reference compounds were performed in Systems 3 or 4 using the RP-HPLC method with gamma or UV/Vis detection, respectively. Radioactivity of the collected samples were measured using Wizard^2^ 2-Detector Gamma Counter (PerkinElmer) and/or Atomlab 500 Dose Calibrator (BIODEX). Electrospray ionization mass spectrometry analyses (ESI-MS) were performed to confirm the presence of the proper compounds.

**System 3**: RP-HPLC semi-preparative Phenomenex Jupiter Proteo 90Å column, 4 μm, 250 × 10 mm, with Jupiter Proteo precolumn, 20 × 2.1 mm, gamma or UV/VIS detection (220 nm), solvent A: acetonitrile with 0.1% TFA (*v/v*), solvent B: water with 0.1% TFA (*v/v*), gradient elution: 0–20 min 20 to 80% A, 20–30 min 80% A, 30–32 min 80 to 20% A, flow 2 mL/min.

**System 4**: RP-HPLC analytical Phenomenex Jupiter 4u Proteo 90Å column, 4 μm, 250 × 4.6 mm, gamma or UV/VIS detection (220 nm), solvent A: acetonitrile with 0.1% TFA (*v/v*), solvent B: water with 0.1% TFA (*v/v*), gradient elution: gradient elution: 0–20 min 1 to 50% A, 20–25 min 50 to 95% A, 25–31 min 95% A, 31–35 min 95 to 1% A. flow 1 mL/min.

#### 2.2.2. Syntheses

Synthesis of conjugates **1** and **2**

**Figure 1:**
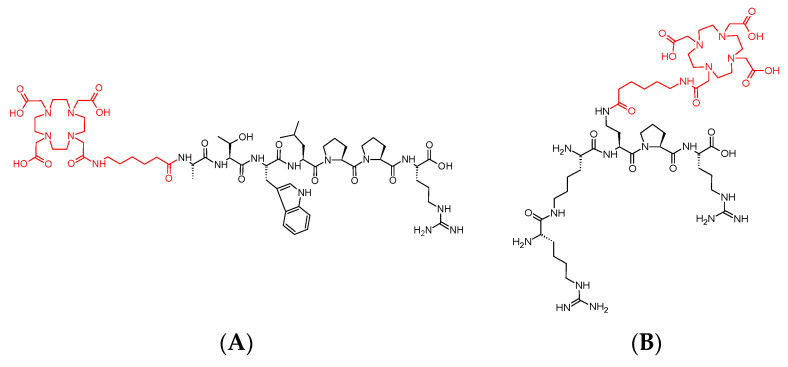
Chemical structure of molecules: (**A**) DOTA-Ahx-A7R (conjugate **1**); (**B**) Lys(hArg)-Dab(Ahx-DOTA)-Pro-Arg (conjugate **2**). The DOTA chelator and Ahx linker that were attached to the parent compounds (in black) are shown in red.

The synthesis of conjugates DOTA-Ahx-A7R (**1**, Figure 1A) and Lys(hArg)-Dab(Ahx-DOTA)-Pro-Arg (**2**, Figure 1B), based on A7R (Figure 1A black) as a parent peptide and its shorter analogue Lys(hArg)-Dab-Pro-Arg (Figure 1B black), respectively, were carried out manually, on the preloaded Fmoc-Arg(Pbf)-Wang resin with a capacity of 0.32 mmol/g (0.48 mmol scale), following the Fmoc chemistry. Coupling of 3 eq. amino acids was done using 3 eq. DIC and 3 eq. HOBt in DMF (3 mL) [49]. Completion of coupling was checked using a Kaiser [50] or chloranil test [51]. Fmoc deprotection step was done using 30% piperidine in DMF. Guanylation reaction was performed for 4 days using di-Boc-S-methylisothiourea (3 eq. in 3 mL DCM) [52]. The Alloc deprotection step was done using tetrakis(triphenylphosphine)palladium(0) (0.06 mmol; 0.25 eq.) in the presence of 24 eq. PhSiH_3_ (5.76 mmol) in DCM [53]. The coupling of the chelator was performed using the active ester method with 1 eq. DOTA-tris(tBu)-NHS and 3 eq. Et_3_N for about 20 h. 

The cleavage of the final conjugates were performed using the mixture of TFA:PhOH:H_2_O:TIPS (88:5:5:2, *v/v*/*v/v*) for 2 h. Crude conjugates were precipitated by a dropwise addition into a cold diethyl ether, and then were purified using semi-preparative KNAUER RP-HPLC in System 1a for conjugate **1** and System 2a for conjugate **2**. Purified conjugates were analyzed by mass spectrometry method (ESI-MS). 

Preparation of [^68^Ga]Ga-DOTA-Ahx-A7R (^68^Ga-1) and [^68^Ga]Lys(hArg)-Dab(Ahx-DOTA-Ga)-Pro-Arg (^68^Ga-2) radioconjugates

[^68^Ga]Ga-DOTA-Ahx-A7R (**^68^Ga-1**) and [^68^Ga]Lys(hArg)-Dab(Ahx-DOTA-Ga)-Pro-Arg (**^68^Ga-2**) radioconjugates were synthesized according to the following procedure: into a vial containing about 10–20 nmol of lyophilized conjugate **1** or conjugate **2,** we added 300–400 μL of 0.2 M acetate buffer (pH 4.5), and 200–300 μL of the [^68^Ga]GaCl_3_ (10–30 MBq) solution from the ^68^Ge/^68^Ga generator. The reaction mixture at pH 3.0 was heated for 10 min at 95 °C. The resulting radioconjugates were then purified by RP-HPLC in System 3 with gamma detection for radioconjugate **^68^Ga-1** (RCY 93.3 ± 0.3%, *n* = 4, molar activity 1.2 MBq/nmol, pH 3.0) and in System 4 with gamma detection for radioconjugate **^68^Ga-2** (RCY 91.5 ± 0.8%, *n* = 4, molar activity 1.2 MBq/nmol, pH 3.0). Pure fractions of **^68^Ga-1** and**^68^Ga-2** radioconjugates were evaporated under N_2_ and dissolved on PBS. 

Preparation of [^177^Lu]Lu-DOTA-Ahx-A7R (^177^Lu-1) and [^177^Lu]Lys(hArg)-Dab(Ahx-DOTA-Lu)-Pro-Arg (^177^Lu-2) radioconjugates

[^177^Lu]Lu-DOTA-Ahx-A7R (**^177^Lu-1**) and [^177^Lu]Lys(hArg)-Dab(Ahx-DOTA-Lu)-Pro-Arg (**^177^Lu-2**) radioconjugates were synthesized according to the following procedure: into a vial containing about 2.5–20 nmol of lyophilized conjugate **1** or conjugate **2**, we added 200–300 μL of 0.2 M acetate buffer (pH 4.5), 150–200 μL H_2_O, and 5–15 μL of the [^177^Lu]LuCl_3_ (1–15 MBq) solution. In some cases, we also added 0.5–2.0 µL 0.1 M HCl to obtain a desired pH. The reaction mixture at pH 4.5 was heated for 10 min at 95 °C. The resulting conjugates were then purified by RP-HPLC in System 3 with gamma detection for radioconjugate **^177^Lu-1** (RCY 95.5 ± 1.2%, *n* = 4, molar activity 0.3 MBq/nmol, pH 4.5) and in System 4 with gamma detection for radioconjugate **^177^Lu-2** (RCY 96.2 ± 2.6%, *n* = 4, molar activity 0.3 MBq/nmol, pH 4.5). Pure fractions of **^177^Lu-1** and **^177^Lu-2** radioconjugates were evaporated under N_2_ and dissolved on PBS.

All radioconjugates were purified before the use in further experiments, namely, the lipophilicity and stability studies.

Preparation of cold reference compounds Ga/Lu-DOTA-Ahx-A7R (Ga/Lu-1) and Lys(hArg)-Dab(Ahx-DOTA-Ga/Lu)-Pro-Arg (Ga/Lu-2)

To verify the identity of the **^68^Ga-1**, **^177^Lu-1**, **^68^Ga-2**, and **^177^Lu-2** radioconjugates, the analogues with stable gallium and lutetium isotopes under the same reaction conditions were synthesized and analyzed by the RP-HPLC method (System 3 or 4 with UV/Vis detection) and ESI-MS methods. 

Ga-DOTA-Ahx-A7R (**Ga-1**) and Lys(hArg)-Dab(Ahx-DOTA-Ga)-Pro-Arg (**Ga-2**) cold reference compounds were synthesized according to the following procedure: into a vial containing approximately 70 nmol of conjugate **1** or **2** dissolved in 300 μL of 0.2 M acetate buffer (pH 4.5), we added the 60 μL of 1.34 mg/mL GaCl_3_ solution in 0.065 M HCl. The reaction mixture at pH 4.0 was heated for 10 min at 95 °C. The reaction progress was checked by RP-HPLC in System 3 for **Ga-1** and in System 4 for **Ga-2**.

Lu-DOTA-Ahx-A7R (**Lu-1**) and Lys(hArg)-Dab(Ahx-DOTA-Lu)-Pro-Arg (**Lu-2**) cold reference compounds were synthesized according to the following procedure: to a vial containing approximately 70 nmol of conjugate **1** or **2** dissolved in 600 μL of 0.2 M acetate buffer (pH 4.5), we added the 3.5 μL of concentrated LuCl_3_ solution in 14 M HCl. The reaction mixture at pH 4.0 was heated for 10 min at 95 °C. The reaction progress was checked by RP-HPLC in System 3 for **Lu-1** and in System 4 for **Lu-2**.

#### 2.2.3. Physicochemical Properties Study of the Radioconjugates

All studies of the physicochemical properties of the synthesized radiopreparations were carried out using radioconjugates previously isolated from the reaction mixture.

Radioconjugates lipophilicity test

The lipophilicity (L) of tested radiocompounds, defined as the decimal logarithm (logP) of the partition coefficient (P) of the compound between the two immiscible phases, was determined in the system PBS solution (aqueous phase, polar phase, pH 7.4) and *n*-octanol (organic, non-polar phase) according to the following formula:

$$L=\mathrm{logP}=\log\frac{A_{o}}{A_{w}}$$

where $A_{o}$—organic phase radioactivity; $A_{w}$—aqueous phase radioactivity.

Radioactivity of both phases, resulting from the concentration of the tested radiocompound in each of them, was determined by measuring the gamma radiation with a Wizard counter in three independent experiments. 

Before starting the research, both liquid phases were saturated with each other to avoid errors resulting from the mutual solubility of both liquids. The partition coefficient (P) result is shown as the mean ± SD. After the end of the experiment, we performed RP-HPLC analysis of the aqueous phase to confirm the tested radioconjugate remained intact during the experiment.

Radioconjugates stability tests

In accordance with requirements for potentially novel radiopharmaceuticals, we tested newly designed radiopreparations for their stability in solutions that act as human body fluids and also in human serum [54,55].

Stability studies in PBS buffer, cysteine and histidine

Stability studies in PBS buffer were performed in order to investigate the possible influence of buffer components on the radiocompound decomposition process. We isolated **^68^Ga-1**, **^177^Lu-1**, **^68^Ga-2**, and **^177^Lu-2** radioconjugates from the reaction mixture and incubated each in PBS buffer. After about 3 h, we analyzed aliquots by RP-HPLC in System 3 with gamma detection for **^68^Ga-1** and **^177^Lu-1** and in System 4 with gamma detection for **^68^Ga-2** and **^177^Lu-2**.

Stability studies of the isolated from the reaction mixture **^68^Ga-1**, **^177^Lu-1**, **^68^Ga-2,** and **^177^Lu-2** radioconjugates in solutions containing excess amounts of strongly competing natural ligands with chemically reactive groups, e.g., -NH_2_, -SH, and -COOH (the so-called challenge experiments). For this purpose, each radioconjugate was incubated at 37 °C in 1 mM cysteine (Cys) or histidine (His) solutions in PBS buffer (the ligand concentration was about 1000 times higher than the concentration of tested radioconjugate). After the specified incubation time, which was up to 4 h for the ^68^Ga-radiocompounds and up to 6 days for the ^177^Lu-radiocompounds, we analyzed solutions by RP-HPLC in System 3 with gamma detection for **^68^Ga-1** and **^177^Lu-1** and in System 4 with gamma detection for **^68^Ga-2** and **^177^Lu-2**.

Stability studies in human serum

Stability studies of the **^68^Ga-1**, **^177^Lu-1**, **^68^Ga-2**, and **^177^Lu-2** radioconjugates in human serum (HS) were performed according to the following procedure: into a vial containing 900 µL of HS we added 100 µL of the tested radioconjugate and incubated the mixture at 37 °C. After the specified incubation times (10 min–4.5 h for ^68^Ga-radiocompounds and 10 min–4 days for ^177^Lu-radiocompounds), we withdrew the aliquots, mixed with ethanol to precipitate the proteins, and centrifuged (14,000 rpm, 5–15 min) to separate the protein compounds from the supernatant. The gamma radioactivity of the obtained precipitate and supernatant was then measured using a Wizard detector. Using the formula below, the amount of radioconjugate remaining in the supernatant (and also bound by serum protein components) was calculated.

$$\frac{\sum \;\mathrm{liquid}\;\mathrm{phase}\;\mathrm{radioactivity}}{\sum \;\mathrm{liquid}\;\mathrm{phase}\;\mathrm{radioactivity}+\sum \;\mathrm{percipitate}\;\mathrm{radioactivity}\;}\times 100\%$$

We also analyzed the supernatant at each time by RP-HPLC in System 3 with gamma detection for **^68^Ga-1** and **^177^Lu-1** and System 4 with gamma detection for **^68^Ga-2** and **^177^Lu-2** to check whether the studied radiocompound remained unchanged during the experiment.

## 3. Results

### 3.1. Syntheses of Conjugates **1** and **2**

**Scheme 1:**
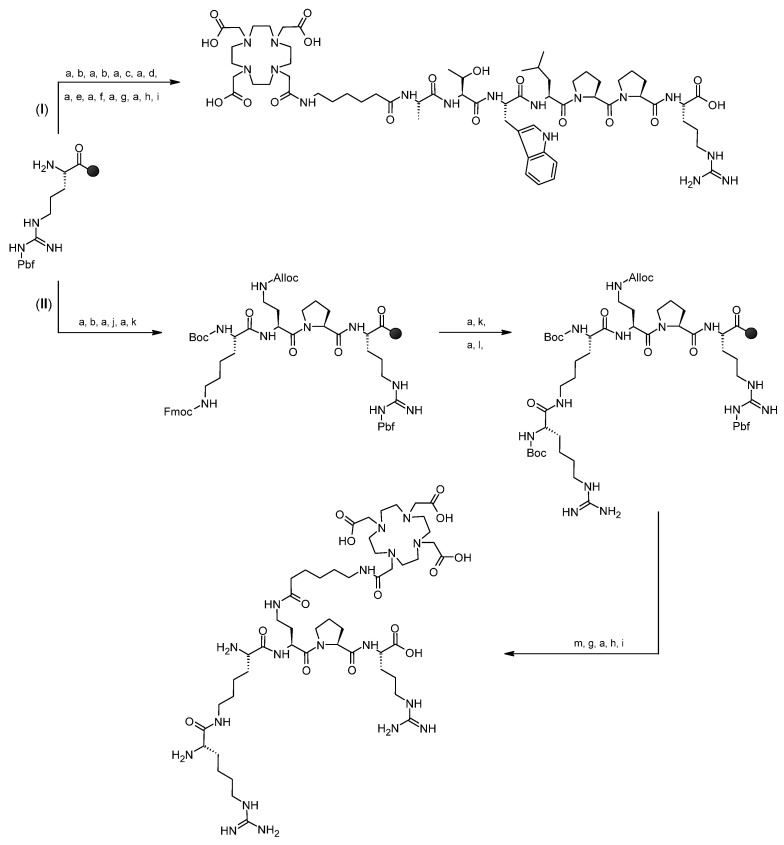
Synthesis strategy of conjugate **1** (path I) and **2** (path II) by SPPS method. a—30% piperidine in DMF; b—Fmoc-Pro-OH:DIC:HOBt; c—Fmoc-Leu-OH:DIC:HOBt; d—Fmoc-Trp(Boc)-OH:DIC:HOBt; e—Fmoc-Thr(tBu)-OH:DIC:HOBt; f—Fmoc-Ala-OH:DIC:HOBt; g—Fmoc-Ahx-OH:DIC:HOBt; h—DOTA-tris(tBu)-NHS:triethylamine; i—TFA/PhOH/H_2_O/TIPS; j—Fmoc-L-Dab(Alloc)-OH:DIC:HOBt; k—Boc-Lys(Fmoc)-OH:DIC:HOBt; l—di-Boc-S-methylisothiourea:n-butylamine:DCM; m—phenylsilane:tetrakis(triphenylphosphine)-palladium:DCM.

The synthesis of conjugates **1** and **2** (Scheme 1) was carried out by Solid Phase Peptide Synthesis (SPPS) on the preloaded Fmoc-Arg(Pbf)-Wang resin following the Fmoc chemistry according to the standard coupling DIC/HOBt protocol. In the case of synthesis of conjugate **1**, after building a linear peptide on the resin and coupling of the Ahx spacer, the chelator DOTA was attached by the active ester method using DOTA-tris(tBu)-NHS. 

The synthesis of conjugate **2** required a few additional steps, as shown in Scheme 1. After the synthesis of fully protected, linear tripeptide (Fmoc-Dab(Alloc)-Pro-Arg(Pbf)-Wang), we attached the N-terminal lysine using Boc-Lys(Fmoc), which enabled the attachment of another Boc-Lys(Fmoc) to the side chain after removal of the Fmoc group from the epsilon amino group. After Fmoc deprotection of the epsilon amino group of the second lysine, we carried out the guanylation reaction for 4 days to lead to hArg creation using di-Boc-S-methylisothiourea, until the Kaiser test was negative. Next, after Alloc deprotection of the amino group of the side chain of the Dab residue, we coupled Fmoc-Ahx. Following the coupling sequence, Fmoc deprotection was performed as the next step, and then the DOTA chelator was attached by the active ester method using DOTA-tris(tBu)-NHS. The cleavage of the synthetized compounds from the resin was performed using the TFA:PhOH:H_2_O:TIPS mixture simultaneously removing all Boc protections. We purified the crude compounds by RP-HPLC and analyzed by ESI-MS (Table 1).

### 3.2. Syntheses of Radioconjugates

**Figure 2:**
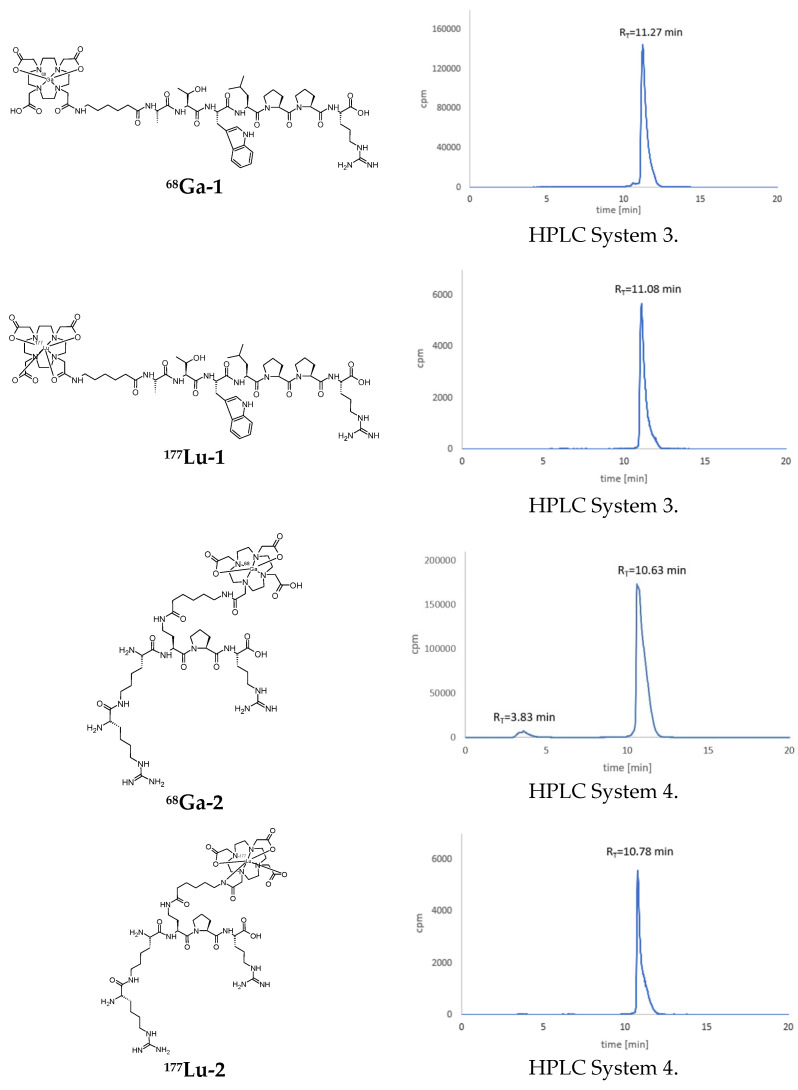
The chemical structures (**left**) and RP-HPLC radiochromatograms (**right**) of the **^68^Ga-1**, **^177^Lu-1**, **^68^Ga-2**, and **^177^Lu-2** radioconjugates.

All obtained radiocompounds were formed with high radiochemical yield (>90% for **^68^Ga-1** and **^68^Ga-2**, and >95% for **^177^Lu-1** and **^177^Lu-2**) and high radiochemical purity (>90%). The chemical formulas of the **^68^Ga-1**, **^177^Lu-1**, **^68^Ga-2**, and **^177^Lu-2** radioconjugates as well as the RP-HPLC radiochromatograms of the labeling reaction mixtures are presented in Figure 2. The statistical data of the radiochemical yield (RCY) and radiochemical purity (RCP) (presented as the mean ± SD, *n* = the number of repetitions) of the obtained **^68^Ga-1**, **^177^Lu-1**, **^68^Ga-2**, and **^177^Lu-2** radioconjugates are presented in Table 2.

### 3.3. Synthesis of Cold References Compounds

**Figure 3:**
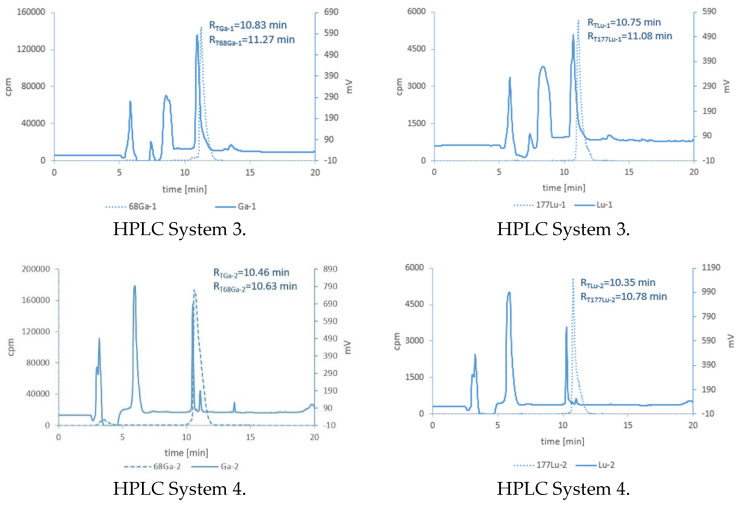
RP-HPLC chromatograms of the reaction mixtures of the cold reference compounds **Ga-1**, **Lu-1**, **Ga-2**, and **Lu-2**—solid lines—and their corresponding radioconjugates **^68^Ga-1**, **^177^Lu-1**, **^68^Ga-2**, and **^177^Lu-2**—dotted lines.

RP-HPLC analyses of the reaction mixtures of the cold reference compounds are presented in Figure 3. The compounds characterized with R_T_ values as 10.83 min, 10.75 min, 10.46 min, and 10.35 min were isolated from the reaction mixture and identified by ESI-MS as Ga-DOTA-Ahx-A7R (**Ga-1**), Lu-DOTA-Ahx-A7R (**Lu-1**), Lys(hArg)-Dab(Ahx-DOTA-Ga)-Pro-Arg (**Ga-2**), and Lys(hArg)-Dab(Ahx-DOTA-Lu)-Pro-Arg (**Lu-2**), respectively. The R_T_ values of these reference compounds coincided with the corresponding retention times of the appropriate radiocompounds (**^68^Ga-1**, **^177^Lu-1**, **^68^Ga-2**, and **^177^Lu-2**; Figure 3), which confirmed the stability of both the A7R and Lys(hArg)-Dab-Pro-Arg molecules under these synthesis conditions and confirmed that we obtained the desired radiocompounds.

The results of ESI-MS analyses confirming the cold reference compounds were obtained are shown in Table 3.

### 3.4. Lipophilicity Studies

Lipophilicity is one of the most important physicochemical parameters affecting the absorption, distribution, metabolism, and excretion of drug molecules in the organism, the so-called ADME profile [56,57,58,59]. Preparations characterized by high lipophilicity show a high affinity for fats and a low affinity for water; they are also able to effectively penetrate cell membranes. From the point of view of pharmacology, lipophilicity is an important prognostic factor for drugs and other medical preparations to predict toxic activity and to characterize biological activity, the ability to accumulate in organisms, and the metabolism of substances [60,61,62,63,64,65]. The lipophilicity values obtained for tested radioconjugates **^68^Ga-1**, **^177^Lu-1**, **^68^Ga-2**, and **^177^Lu-2** (presented as the mean ± SD) are shown in Table 4. The main reason for the low lipophilicity value is that the tested radiocompounds are based on peptides and peptidomimetics, which are usually characterized by a low lipophilicity value. Moreover, conjugation of the macrocyclic DOTA chelator with the biomolecule also results in a lower lipophilicity value of the conjugate compared to the lipophilicity of the biomolecule itself.

Radiochromatograms of the aqueous phases after the lipophilicity tests of the radiopreparations showed, in all cases, single peaks at R_T_ values that corresponded to the tested radiocompounds, which proves the stability of the radiopreparations during the experiment.

### 3.5. Stability Studies of Radioconjugates in PBS Buffer, Cysteine and Histidine

Stability tests of all radioconjugates incubated in PBS solution for about 3 h showed no decomposition of radioconjugates. In all the RP-HPLC radiochromatograms only single peaks were recorded, with R_T_ values that corresponded to the appropriate radioconjugate, which confirms the stability of radioconjugates under such conditions.

### 3.6. Stability Tests for **^68^Ga-1** and **^68^Ga-2** Radioconjugates

**Figure 4:**
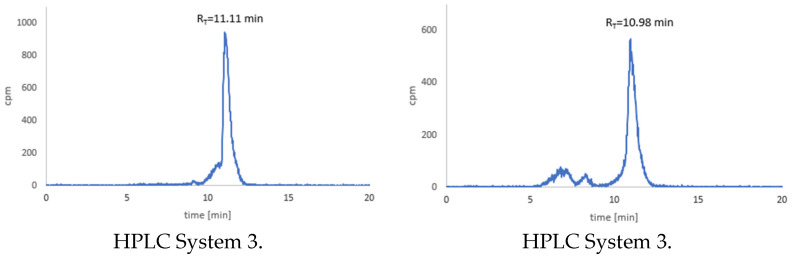
RP-HPLC radiochromatograms (System 3) of the stability studies of the **^68^Ga-1** radioconjugate in Cys (**left**, RCP 95.2 ± 1.5%, *n* = 3) and His (**right**, RCP 76.2 ± 2.7%, *n* = 3) solutions recorded after 3.5 h of incubation.

Stability tests of the **^68^Ga-1** radioconjugate in the Cys (Figure 4 left) and His (Figure 4 right) solutions showed satisfactory stability in the period of about 3 h, corresponding to the three half-lives of the radionuclide gallium-68. In the RP-HPLC radiochromatograms recorded after 3 h, we observed only traces of the products of radioconjugate decomposition (Figure 4). 

**Figure 5:**
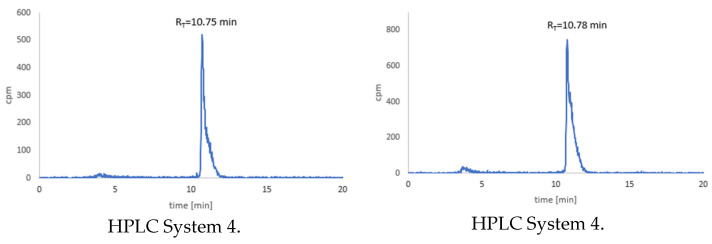
RP-HPLC radiochromatograms (System 4) of the stability studies of the **^68^Ga-2** radioconjugate in Cys (**left**, RCP 98.1 ± 1.2%, *n* = 3) and His (**right**, RCP 95.3 ± 2.0%, *n* = 3) solutions recorded after 3.5 h of incubation.

Similarly, stability studies of the **^68^Ga-2** radioconjugate in Cys (Figure 5 left) and His (Figure 5 right) solutions carried out over the same time interval demonstrated the complete stability of the radioconjugate.

### 3.7. Stability Tests for **^177^Lu-1** and **^177^Lu-2** Radioconjugates

**Figure 6:**
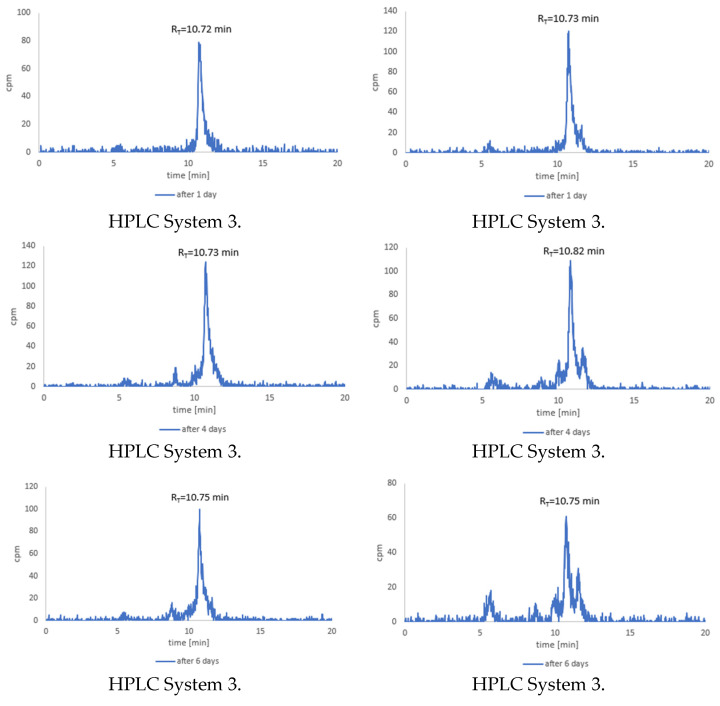
RP-HPLC radiochromatograms (System 3) of the stability studies of the **^177^Lu-1** radioconjugate in Cys (**left**, after 1 day RCP 98.2 ± 2.5%, *n* = 3; after 4 days RCP 94.5 ± 3.0, *n* = 3; after 6 days RCP 93.0 ± 3.6%, *n* = 3) and His (**right**, after 1 day RCP 95.6 ± 2.5%, *n* = 3; after 4 days RCP 82.2 ± 3.2%, *n* = 3; after 6 days RCP 64.5 ± 4.1%, *n* = 3) solutions recorded after different time intervals.

Stability studies of the radioconjugates containing lutetium-177 radionuclide in Cys and His solutions were carried out in the period of about 6 days, corresponding to the one half-live of the radionuclide lutetium-177. Incubation of the **^177^Lu-1** radioconjugate in Cys and His solutions for a longer period, more than 4 days, showed slow degradation of this radiopreparation in both solutions (Figure 6). The radioconjugate was completely stable for 1 day, but after 6 days, traces of the products of radioconjugate decomposition were already visible, and the degradation of the radioconjugate was noticeably higher during incubation in the His solution (Figure 6 right).

**Figure 7:**
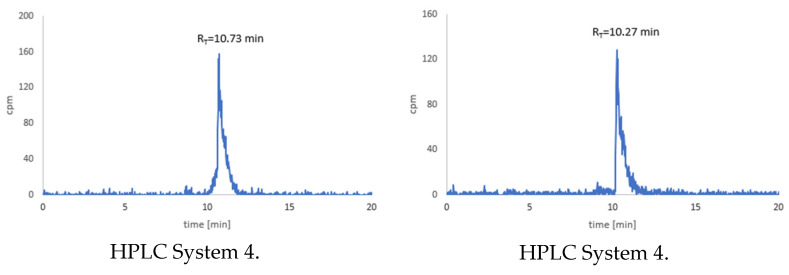
RP-HPLC radiochromatograms (System 4) of the stability studies of the **^177^Lu-2** radioconjugate in Cys (**left**, RCP 98.3 ± 2.5%, *n* = 3) and His (**right**, RCP 98.3 ± 2.8%, *n* = 3) solutions recorded after 6 days.

In contrast, the **^177^Lu-2** radioconjugate proved to be completely stable in both solutions throughout the incubation period (Figure 7).

### 3.8. Stability Studies of Radioconjugates in Human Serum

**Figure 8:**
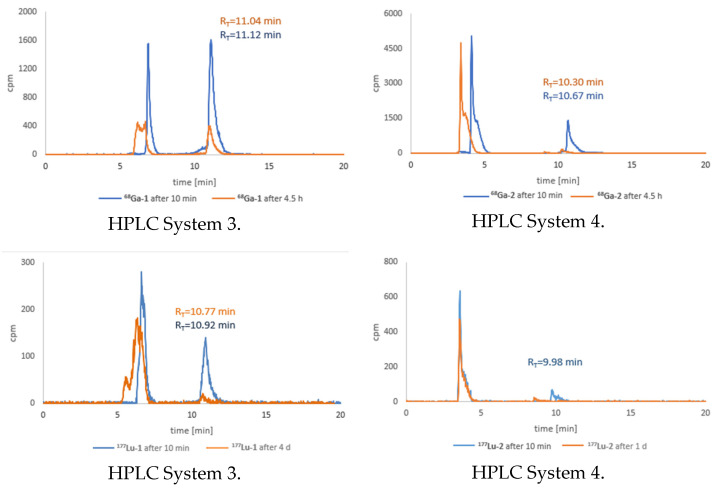
RP-HPLC radiochromatograms illustrating the results of the stability tests of the **^68^Ga-1** and **^177^Lu-1** (**left**, System 3) and **^68^Ga-2**, **^177^Lu-2** (**right**, System 4) radioconjugates in human serum recorded after different time intervals.

Radiochromatograms illustrating the results of the stability tests of the **^68^Ga-1**, **^177^Lu-1** and **^68^Ga-2**, **^177^Lu-2** radioconjugates in human serum are shown in Figure 8. The statistical data of the radiochemical purity (presented as the mean ± SD, *n* = the number of repetitions) of the obtained **^68^Ga-1**, **^177^Lu-1**, **^68^Ga-2**, and **^177^Lu-2** radioconjugates in the human serum stability tests are presented in Table 5.

As can be seen, all radioconjugates were unstable in HS due to biodegradation of the A7R peptide and Lys(hArg)-Dab-Pro-Arg peptidomimetic by endogenous enzymes. After 10 min of incubation, the radiochromatograms already showed additional peaks corresponding to the enzymatic degradation products. During incubation, the height of these additional peaks increased, and the peak heights corresponding to the tested radioconjugates decreased. After about 4 h in the case of the **^68^Ga-1** and **^68^Ga-2** radioconjugates and about 4 days in the case of the **^177^Lu-1** and **^177^Lu-2** radioconjugates, the peaks corresponding to the tested radiopreparations were often hardly visible, indicating almost complete biodegradation of the radioconjugates. Radioactivity measurements of the precipitated protein serum components and the supernatants indicated that, in the case of the **^68^Ga-1** and **^177^Lu-1** radioconjugates, about 15% of the radiocompounds was bound to the protein serum components. In the case of the **^68^Ga-2** and **^177^Lu-2** radioconjugates, about 28% of the radiocompounds was bound with protein serum components.

## 4. Discussion

The aim of the present study was to synthesize and to characterize the physicochemical properties of radiolabeled A7R peptide and Lys(hArg)-Dab-Pro-Arg peptidomimetic for potential application as diagnostic or therapeutic receptor radiopharmaceuticals. According to the literature data, the heptapeptide A7R is known to be an effective antagonist of the VEGF-A_165_ binding with NRP-1 and to show in vivo anti-angiogenic properties [35], whereas the Lys(hArg)-Dab-Pro-Arg peptidomimetic is much more active (IC_50_ = 0.2 μM) than the heptapeptide A7R (IC_50_ = 5.9 μM [42]), with a stability half-life in human serum that is nearly 2 days [41,43]. To be able to synthesize radioconjugates, we attached a DOTA chelator to the initial compounds—A7R or its shorter analogue—via an Ahx linker.

All the designed radioconjugates were synthesized with high radiochemical yield and high radiochemical purity (Figure 2 right), and the methods for their synthesis were relatively simple and cheap. Identity of the radioconjugates was confirmed by ESI-MS analysis of their cold reference compounds. 

All radioconjugates turned out to be highly hydrophilic compounds with logP values in the range of −3.40 to −4.57 (Table 4). Moreover, the radioconjugates with Ga-68 turned out to be visibly less lipophilic due to a free carboxyl group of the DOTA chelator. These values were much lower than those preferred for radiopharmaceuticals, in the ranges of 1 to 4 and 1.5 to 2.5, which are suitable for crossing the blood–tissue [66] and blood–brain barriers [67], respectively. However, the ability to cross the blood–tissue barrier is not a crucial parameter in this case due to the presence of VEGF_165_ in the endothelium, the single layer of squamous endothelial cells that lines the interior surface of blood vessels, and its ability to interact directly with blood.

Radioconjugates **^68^Ga-1** and **^68^Ga-2** and radioconjugate **^177^Lu-2** were sufficiently stable in solutions acting as human body fluids, PBS buffer, and Cys or His solutions. However, the stability of the **^177^Lu-1** radioconjugate that contained therapeutic radionuclide Lu-177 and based on the A7R peptide turned out to be rather insufficient. After 6 days of incubation, which is just one half-life of the radionuclide Lu-177, the radioconjugate decomposition products were already visible (Figure 6). Because this is a potential therapeutic radiopharmaceutical containing radionuclide with a half-life of 6.65 days, such stability in these solutions is rather low.

Stability studies of the radioconjugates in human serum showed relatively rapid decomposition of all radiocompounds due to enzymatic biodegradation of A7R peptide and Lys(hArg)-Dab-Pro-Arg peptidomimetic (Figure 8). Comparing the RP-HPLC radiochromatograms recorded after a 10 min incubation, it is apparent that the radioconjugates **^68^Ga-2** and **^177^Lu-2** were more readily biodegraded in HS compared to the radioconjugates **^68^Ga-1** and **^177^Lu-1**. In the case of **^177^Lu-1** radiopreparation after 4 days of incubation, the radioconjugate was still visible in the sample, while in the case of **^177^Lu-2** after 1 day of incubation, only traces of radioconjugate were present in the sample. Moreover, during the incubation of **^68^Ga-1** and **^177^Lu-1** in HS, about 15% of the radiopreparations were bound to the serum peptide components, while in the case of radioconjugates **^68^Ga-2** and **^177^Lu-2**, almost twice as much, about 28%. This difference can be explained by the less-polar nature of the A7R (Ala^1^-Thr^2^-Trp^3^-Leu^4^-Pro^5^-Pro^6^-Arg^7^-OH) peptide and the polar nature of the Lys^1^(hArg)-Dab^2^-Pro^3^-Arg^4^ peptidomimetic. The former compound had three reactive functional groups—the guanidine group of Arg^7^, the amine group of Ala^1^, and the carboxyl group of Arg—while the peptidomimetic had six reactive functional groups—the three amino groups of Lys^1^, hArg, and Dab^2^, the two guanidine groups of hArg and Arg^4^, and one carboxyl group of Arg^4^. These reactive groups, depending on the pH and under the influence of external conditions, can assume, respectively, positive or negative charges, and interact with the protein components of the human serum. Despite the fact that one amine group was used to attach the DOTA chelator via the Ahx linker in both conjugates, the radioconjugates produced by the labeling reactions of conjugate **1** and **2** should still differ in charge, polarity, and chemical reactivity because of differences in the physicochemical properties of their parent compounds, the A7R peptide and Lys(hArg)-Dab-Pro-Arg peptidomimetic.

Based on the above data, it should be assessed that, unfortunately, radioconjugates **^68^Ga-2** and **^177^Lu-2** that were based on the more biologically active biomolecule, Lys(hArg)-Dab-Pro-Arg peptidomimetic, were less stable in HS than those based on the A7R peptide, **^68^Ga-1** and **^177^Lu-1**. Such a low stability of **^68^Ga-2** and **^177^Lu-2** was initially surprising because, according to the literature data, the determined half-life of the Lys(hArg)-Dab-Pro-Arg peptidomimetic in HS was nearly 2 days (41 h), and the period of its complete decomposition in HS estimated from the graph was over 4 days [43]. As determined from our experiments, the period of almost complete enzymatic biodegradation of radioconjugate **^177^Lu-2**, resulting from enzymatic biodegradation of the Lys(hArg)-Dab-Pro-Arg peptidomimetic, was less than 1 day. These two seemingly contradictory results were due to the different conditions of the stability studies. In the first case, the peptidomimetic concentration in the sample was 0.9 µmol/mL, which allowed us to detect the biodegradation using the UV/VIS detection [43]. In the second case, the peptidomimetic concentration was equal to the concentration of the radioconjugate and was incomparably lower, i.e., about 2 nmol/mL; experiments with such a low radioconjugate concentration were possible due to using gamma detection. Consequently, in the first case, the test was carried out with saturation of the enzymes present in HS sample, while in the second case, the amount of the peptidomimetic was much smaller than the amount of enzyme. Hence, in the first case, intact peptidomimetic was visible in the sample for about 4 days, while this period was only 1 day in the present study.

To conclude, our study showed that all radioconjugates obtained in the presented work were not sufficiently stable in HS, and so they unfortunately do not fully meet the requirements for radiopharmaceuticals. Nevertheless, the structure of the peptidomimetics can be modified easily, which makes it possible to design a new formulation that is more stable in HS. In the near future, we plan to modify them by using D-amino acids or replacing one or more peptide bonds with their bioisosteres (e.g., N-methyl peptide bond, or reduced bond -CH_2_-NH-). For radiopreparations sufficiently stable in human serum and suitable for in vivo applications, we plan more advanced biological tests (using NRP-1 overexpressing cell lines) and finally tests on living organisms (a biodistribution study in mice or rats).

## Figures and Tables

**Table 1:** Results of the ESI-MS analyses of synthesized conjugates **1** and **2**.

Conjugate	R_T_ (min) (RP-HPLC System)	Signal Found (m/z)	Signal Calculated (m/z)
**1**	10.9 (1)	[M+3H]^3+^: 447.5	[M+3H]^3+^: 447.0
		[M+2H]^2+^: 670.7	M+2H]^2+^: 670.0
		[M−2H]^2−^: 668.6	[M−2H]^2−^:668.0
		[M−H]^−^: 1337.9	[M−H]^−^: 1337.9
**2**	9.3 (2)	[M+4H]^4+^: 293.2	[M+4H]^4-^: 293.0
		[M+3H]^3+^: 390.6	[M+3H]^3+^: 390.3
		[M+2H]^2+^: 585.4	[M+2H]^2+^: 585.0
		[M−H]^−^: 1167.7	[M−H]^−^: 1167.0

**Table 2:** Results of radiochemical yield and radiochemical purity of the synthesized **^68^Ga-1**, **^177^Lu-1**, **^68^Ga-2**, and **^177^Lu-2** radioconjugates.

Radioconjugate	RCY ± SD, *n* = 4 (%)	RCP ± SD, *n* = 4 (%)
**^68^Ga-1**	93.3 ± 0.3	97.1 ± 0.9
**^177^Lu-1**	95.5 ± 1.2	99.8 ± 0.9
**^68^Ga-2**	91.5 ± 0.8	93.4 ± 0.3
**^177^Lu-2**	96.2 ± 2.6	99.3 ± 1.9

**Table 3:** Results of ESI-MS analyses of the synthesized cold reference compounds **Ga-1**, **Lu-1**, **Ga-2**, **Lu-2**.

Compound	R_T_ (min) (RP-HPLC System)	Signal Found (m/z)	Signal Calculated (m/z)
**Ga-1**	10.83 (3)	[M]^+^: 1405.60 and 1407.61	[M]^+^: 1405.64 and 1407.64
**Lu-1**	10.75 (3)	[M+H]^+^: 1511.63	[M+H]^+^: 1511.66
**Ga-2**	10.46 (4)	[M]^+^: 1235.60 and 1237.63	[M]^+^: 1235.62 and 1237.62
**Lu-2**	10.35 (4)	[M+H]^+^: 1341.59	[M+H]^+^: 1341.62

**Table 4:** R_T_ and logP values of the tested radioconjugates.

Radiocompound	RP-HPLC System	R_T_ (min)	logP ± SD
**^68^Ga-1**	3	11.27	−3.92 ± 0.03
**^177^Lu-1**	3	11.08	−3.40 ± 0.14
**^68^Ga-2**	4	10.63	−4.57 ± 0.05
**^177^Lu-2**	4	10.78	−3.75 ± 0.08

**Table 5:** Statistical data of the **^68^Ga-1**, **^177^Lu-1**, **^68^Ga-2**, and **^177^Lu-2** radioconjugates radiochemical purity corresponding to the radiochromatograms presented in Figure 8.

Radioconjugate	RCP ± SD, *n* = 3 (%)			
	after 10 min	after 4.5 h	after 1 day	After 4 days
**^68^Ga-1**	65.3 ± 3.7	36.6 ± 1.4	n/a	n/a
**^68^Ga-2**	27.7 ± 2.0	3.6 ± 0.2	n/a	n/a
**^177^Lu-1**	39.2 ± 2.0	---	---	2.6 ± 0.2
**^177^Lu-2**	13.8 ± 1.5	---	2.7 ± 0.1	---

## Data Availability

Not applicable.

## Abbreviations

A7R	Ala-Thr-Trp-Leu-Pro-Pro-Arg-OH peptide
ACN	Acetonitrile
ADME	Absorption, distribution, metabolism, and excretion
Ahx	6-aminohexanoic acid
Alloc	Allyloxycarbonyl group
A_o_	Organic phase radioactivity
A_w_	Aqueous phase radioactivity
Boc COOH	Tert-butyloxycarbonyl group Carboxyl group
Cys	Cysteine
Dab	2,4-Diaminobutyric Acid
DCM	Dichloromethane
DIC	N,N′-Di(propan-2-yl)methanediimine
DMF	N,N-Dimethylformamide
DOTA	2,2′,2″,2‴-(1,4,7,10-Tetraazacyclododecane-1,4,7,10-tetrayl) tetraacetic acid
DOTA-tris(tBu)-NHS	tri-tert-butyl 2,2′,2″-(10-(2-((2,5-dioxopyrrolidin-1-yl)oxy)-2-oxoethyl) -1,4,7,10-tetraazacyclododecane-1,4,7-triyl)triacetate
ESI-MS	Electrospray ionization mass spectrometry analyses
Et_3_N	Triethylamine
E_βmax_	Beta emitter with a maximum energy
Fmoc	9-fluorenylmethoxycarbonyl group
hArg	Homoarginine
HCl	Hydrogen chloride
His	Histidine
HOBt	1H-1,2,3-Benzotriazol-1-ol
HS	Human Serum
IC_50_ L	Half maximal inhibitory concentration Lipophilicity
logP	Decimal logarithm of partition coefficient
m/z	Mass-to-charge ratio
mAb	Monoclonal antibodies
NH_2_	Amino group
NRP-1	Neuropilin-1
P	Partition coefficient
Pbf	Pentamethyl-2,3-dihydrobenzofuran-5-sulfonyl group
PBS PhOH	Phosphate-buffered saline Phenol
PhSiH_3_	Phenylsilane
RP-HPLC	Reverse-Phase High Pressure Liquid Chromatography
R_T_	Retention time
RTKs	Receptor Tyrosine Kinases
SD	Standard deviation
SH	Thiol group
SPPS	Solid Phase Peptide Synthesis
t_1/2_	Half-life time
TFA	Trifluoroacetic acid
TIPS	Tri(propan-2-yl)silane
VEGF	Vascular Endothelial Growth Factor
VEGF-A_165_	Vascular Endothelial Growth Factor-A_165_
VEGFR	Vascular Endothelial Growth Factor Receptor
VEGFR-2	Vascular Endothelial Growth Factor Receptor 2
∑	sigma; operator for summation
